# rna interference and micro rna –oriented therapy in cancer: rationales, promises, and challenges

**DOI:** 10.3747/co.v16i4.486

**Published:** 2009-08

**Authors:** T.F. Duchaine, F.J. Slack

**Affiliations:** * Goodman Cancer Centre and Department of Biochemistry, McGill University, Montreal, QC; † Department of Molecular, Cellular and Developmental Biology, Yale University, New Haven, CT, U.S.A

**Keywords:** Cancer therapy, sirna, mirna, rnai therapy, oncomirs, nucleic acid analogs, tumour-suppressor mirnas

## Abstract

The discovery that rna interference (rnai) and its functional derivatives, small interfering rnas (sirnas) and micro-rnas (mirnas) could mediate potent and specific gene silencing has raised high hopes for cancer therapeutics. The prevalence of these small (18–25 nucleotide) non-coding rnas in human gene networks, coupled with their unique specificity, has paved the way for the development of new and promising therapeutic strategies in re-directing or inhibiting small rna phenomena.

Three strategies are currently being developed:

- *De novo* rnai programming using synthetic sirnas to target the expression of genes
- Strengthening or recapitulation of the physiologic targeting of messenger rnas by specific mirnas
- Sequence-specific inhibition of mi rna functions by nucleic acid analogs

Each strategy, currently being developed both in academia and in industry, holds promise in cancer therapeutics.

## 1. INTRODUCTION

These are very interesting times in the field of gene regulation as it affects development and cancer. Since the late 1990s, the tenets of the “central dogma” have undergone revision as scientists began to appreciate that rnas, by themselves, play key regulatory roles.

Leading the charge in this revolution are the small (18–25 nucleotide) non-coding rnas called “small interfering” rnas (sirnas) and micro-rnas (mirnas), which direct rna interference (rnai) phenomena. The sirnas and mirnas are newly discovered regulatory molecules that control a variety of biologic processes, including development, metabolism, and aging 1–3. The mirnas often bind to complementary sequences in the 3′ untranslated regions of target messenger rnas (mrnas) to repress target gene expression; the sirnas can bind to any complementary sequence in a target mrna. Importantly, functional roles have been demonstrated for mirnas in cancer 4,5, and the potential exists to use similar molecules as cancer therapeutics.

The present review outlines the underlying rationales for considering small rna therapeutics. Although most of the attention has been given to the use of sirnas to inhibit gene function, we provide a broader overview and include novel and emerging strategies that are instead based on mirnas. We highlight the potential of each strategy for cancer treatment and summarize the challenges that need to be overcome to fully harness rnai- and mirna-based therapies.

## 2. THE MOLECULAR BASIS FOR siRNA- AND miRNA-MEDIATED SILENCING

The silencing mediated by rnai and mirna is initiated when the double-stranded rna (dsrna)–specific type iii endonuclease, Dicer, “recognizes” a dsrna substrate. Processing by Dicer and its accessory proteins yields 18- to 25-nucleotide heteroduplex small rnas (either sirnas or mirnas, depending on their origin). The mirnas are usually endogenously transcribed; the sirnas often derive from exogenous sources such as viruses 6,7. One of the strands of the small rna duplex is selected, loaded into Argonaute proteins, and assembled into an effector complex such as the rna-induced silencing complex (risc). The other strand is often cleaved or unwound and degraded 8,9. A key feature for the use of rnai in therapy is that the risc can be directly programmed by exogenous mature sirnas or mirnas. In effect, this programming bypasses cleavage by Dicer and the interferon response triggered by dsrnas larger than 30 bp in mammalian cells 10. Generally, when a sirna fully pairs with a target mrna, the result is rapid and specific cleavage, an activity named “slicer” 11.

In contrast to sirnas, mirnas normally originate from gene-encoded precursor transcripts that are processed into precursor mirnas. Those precursors fold as hairpins and serve as Dicer substrates 12. Embedded within the risc, mature mirnas pair only partially with their mrna targets, thereby directing translation inhibition or reducing the stability of their target 13.

The gene-targeting information is non-symmetrically encoded within mature sirnas or mirnas. The 5′ portion of the mirna at positions 2–7 is thought to encode the most important determinant to direct base-pairing with mrna targets. This portion (called the “core,” “nucleus,” or “seed” sequence) is most commonly conserved; it defines paralogous and orthologous families of mirnas 14. Structural studies show a tight coordination of this 5′ determinant within the Argonaute proteins 15. The 3′ part of the mirna or sirna is more amenable to chemical modifications, some of which occur naturally.

## 3. THE BIOLOGIC IMPLICATIONS OF miRNAS FOR CANCER

To evaluate the potential of small rnas in cancer therapy, an appreciation of how extensively they are tied to the normal cell’s gene regulation networks is essential. In human cells, mirnas are estimated to account for more than 1000 genes (approximately 3% of the total estimated number of genes) and to exert direct negative regulative pressure on more than 30% of the entire coding gene set 14,16. Accordingly, mirnas are known to regulate genes implicated in a wide range of cellular functions in development and homeostasis 17. In *Caenorhabditis elegans,* for example, the first two known mirnas—*lin-4* and *let-7—*control timing of cell differentiation during development. In *let-7* mutants, stem cells in the skin frequently fail to terminally differentiate, electing instead to divide again. Such cellular events are the defining hallmarks of cancer 18.

Because misregulation of genes that control cell proliferation and cell fate often contribute to cancer development, mirnas are unsurprisingly also implicated in cancer biology. Early evidence in humans indicates that mirna loci are associated with chromosome sites that are often deleted or amplified 19, and whose expression is dramatically altered during the onset of cancer 20. Intense current research aims to precisely delineate the functions of mirnas in transformation and tumour growth 21–23. In an increasing number of reports, mirnas are now being shown to be capable of acting as bona fide tumour suppressors or oncogenes, bringing about a major shift in the landscape of cancer research 24.

## 4. siRNA- AND miRNA-BASED THERAPEUTIC STRATEGIES

**FIGURE 1:**
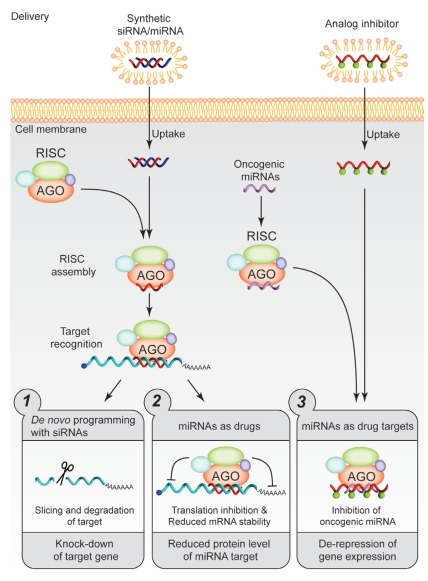
Therapeutic strategies based on “small interfering” rnas (sirnas) and micro-rnas (mirnas). risc = rna-induced silencing complex; ago = Argonaute protein; mrna = messenger rna.

The chemical properties of the various mirnas vary little, and yet, through the sequences that they encode, they direct completely distinct activities. At least three distinct overall strategies were adopted early on for sirna- and mirna-based therapies. Each one aims (reasonably) to exploit the sequence-directed properties of the small rnas:

- First, *de novo* reprogramming of the rnai machinery can be utilized against a gene that has deleterious effects (Figure 1, panel 1).
- Second, a physiologic mi rna–mrna pair could be reproduced by programming the risc using a known, beneficial mirna—for example, a tumour suppressor mirna (Figure 1, panel 2).
- Third, a nonhydrolyzable mimic of an rna target could be generated to effectively sequester deleterious mirnas from their pro-oncogenic functions (Figure 1, panel 3).

### 4.1 Harnessing RNAi: *De Novo* Programming with siRNAs

Perhaps the simplest and the most direct therapeutic strategy involving rnai is to harness and redirect its machinery against a detrimental mrna target. It therefore comes as no surprise that this strategy is by far the most advanced in clinical trials. The sirna-directed targeting of genes is currently being developed as a potential therapy in a great number of diseases 25,26. The keys to this strategy are identification of a functional and effective rnai target and optimization of the targeting process. The strategy can include, but is not limited to, knowing the uniqueness of the sirna’s target sequence in the genome, optimizing its loading into the risc, obtaining a measure of the accessibility of the sirna target site (structure, protein–rna interactions), and possibly even adding tolerated covalent modifications to the sirna. In addition, and common to each rnai- or mirna-based therapy, is the need to deliver the rna molecule or analog to the correct tissue and mrna target (see the “Challenges” subsection).

Many sirna-based therapies are under development, but surprisingly few are directed at oncogenic targets. Among the cancer-directed approaches is the targeting of pleiotrophin, which effectively reduced tumour growth in mouse xenograft models of glioblastoma multiforme 27. Another initiative of interest is the successful targeting of the Ews–Fli1 fusion oncoprotein in mouse models of metastatic Ewing sarcoma 28.

### 4.2 Tilting the Balance: miRNAs As Drugs

A second potential approach in rnai-based cancer therapy is to deliver a mature or engineered mirna precursor to specific biologic compartments to affect its known and physiologic mrna targets. In effect, this strategy aims at the restoration, the strengthening, or the ectopic rewiring of known mirna-target circuitry 29,30. In contrast with the *de novo* programming approach, this strategy relies heavily on prior and extensive characterization of the biologic functions of the mirna of interest. The idea would be

- to know the identity of the physiologic targets of the mirna of interest, and
- to have a good idea of how the associated genes are embedded within their genetic network.

This second aspect is important, considering that mirnas often function as parts of loops (feedback and feed-forward) between gene networks. (For examples, see Li *et al.* 31 and Xu *et al.* 32)

One mirna that fits each of these requirements is *let-7.* Overexpression of *let-7* in a mouse model of lung cancer involving an activated K*-ras* allele suppresses activating oncogenic *ras* mutations just as such overexpression does in *C. elegans* 33,34. Signalling by *ras* plays an important role in radioresistance, and radiotherapy is one of the three primary modalities used in combatting cancer. However, many cancers, including lung cancer, are radioresistant. Because *let-7* represses *ras, let-7* overexpression has been proposed to be a possible cause of radiosensitivity, a hypothesis that indeed proved to be the case in both lung cancer cells and in an *in vivo C. elegans* model 29. These data provided the first evidence that mirnas could potentially augment current cancer therapies.

An even more recent publication reported the use of a similar strategy—named “mirna replacement therapy”—aimed at restoring mir26a expression in hepatocellular carcinoma. When delivered in a mouse model using adeno-associated virus, this mirna was able to suppress proliferation and induce apoptosis, which resulted in inhibition of hepatocellular carcinoma progression 35.

### 4.3 Sequence-Directed Inhibition: miRNAs As Drug Targets

Where the strategies already discussed are based on programming of the risc, a third promising strategy involves inhibition of the endogenous functions of well-characterized mirnas. This inhibition is accomplished by “base-pairing”: using complementary nucleic acid analogs to block the unique signature of small rnas (mainly the “seed”). One major advantage of this strategy is that the constraints required to contact or base-pair with a mirna sequence are likely to be much looser than the requirements to fit within the tightly coordinated binding pocket of the risc and to act as a functional rnai component. These looser constraints in turn allow for greater chemical and covalent modifications to be made to the backbone of the analog inhibitor to change its stability, mirna-inhibiting properties, and even its pharmacokinetics and biodistribution. At this stage, the frontrunners of these modifications are the locked nucleic acids (lnas) 36 2′-*O-*methyl 37,38 and 2′-*O*-methoxyethyl 39,40. In addition to combinations of these modifications, analogs such as the “antagomirs” (mirs) are modified with phosphorothioate substitutions on the backbone and with cholesterol conjugation 36.

A recent and successful example of this overall strategy is the *in vivo* inhibition of mir122 mirna using a 15-nucleotide lna-bearing analog 41. The liver-specific mir122 has been implicated in cholesterol and lipid metabolism and has been linked to replication of hepatitis C virus (hcv) *in vitro* 42. A recent study by Elmen and colleagues is the first to report inhibition of mirnas by analogs *in vivo* in nonhuman primates. Reduction of mature mir122 levels in the liver was accompanied by a substantial and reversible reduction in plasma cholesterol, without inducing any detectable sign of toxicity or tissue damage 41. This work raised the possibility that mir122 could be targeted in chronically infected hcv patients, for example, which could in turn reduce the associated risk of carcinoma. In a broader sense, this work highlights the potential of targeting mirnas using lna analogs in therapy, with low toxicity and high specificity.

Although the use of mirna inhibitors is only starting to emerge as a therapeutic strategy, an important number of studies have reported mirna targeting by analogs in cell culture or mouse models. Successfully targeted and cancer-relevant mirnas include mir16; mir21; mir34a, b, and c; mir29b; mir221/222; and the 17 to approximately 92 cluster of mirnas individually—and the list is rapidly growing (see Stenvang *et al.* 43 and references therein). Considering the role of mirnas in cancer, the low toxicity of lna in primates, and the feasibility of efficient targeting of mirnas *in vivo,* the potential impact of this strategy is immense.

## 5. CHALLENGES

By far the most important challenge faced by researchers developing therapeutic strategies based on rnai is the means to deliver the sirnas, mirnas, or analogs to the correct cells in the organism. Intrinsically, rnas are polar and labile, and their pharmacokinetic properties are completely alien to most of the desired cellular destinations. The stability of the rna molecule can be greatly enhanced by blocking the 2′-hydroxy group of ribonucleotides and modifying the phosphodiester backbone, but delivery must be tailored and optimized for each tissue and cellular target. A few early reports describing direct delivery of naked, unmodified rna molecules to the target tissue appeared successful, one case being sirnas targeting respiratory syncytial virus to the lung 44. In addition, sirna targeting of vascular endothelial growth factors α and 1 in age-related macular degeneration has been reported 45. However, recent work by Kleinman *et al.* called the reasons for these findings into question when those authors reported that at least some of the anti-angiogenesis virtues of the sirna treatment were independent of its sequence 46. Instead, efficacy appeared to depend on the Toll-like receptor 3–triggered induction of the interferon γ response. That study was seen by some as a setback for sirna-based therapies. For others, it meant simply that rnai- or nucleic acid–based therapy in general requires additional fine-tuning to escape the innate immune response. Part of the solution is likely already underway, because an important variety of synthetic material has been established and successfully used for sirna or nucleic acid delivery. The efficient strategies reported range from direct conjugation of cholesterol and nucleic acids or analogs, to complexation or coating with liposomes or polycationic agents. (For a recent and thorough review on this topic, see Whitehead *et al.* 26)

Another important concern for rnai and mirna-based therapy is the unintentional targeting of mrnas, termed “off-targets.” This effect has been well characterized using microarray studies of sirna-transfected cells, in which expression in an important number of genes declined with increased concentration of sirnas 47,48. It is also thought that ectopic expression of mirnas can result in a related effect. Selective pressure normally disfavours the presence of sequences complementary to a mirna “seed” in non-targets and co-expressed mrnas—a phenomenon termed “avoidance” 49. Ectopically expressed or overexpressed mirnas in therapy can therefore potentially find sequences that are avoided under physiologic conditions.

In part, the delivery and off-target issues of sirna-and mirna-based therapies are linked. Ideally, the means to deliver these nucleic acids, or their analogs, to cells would proceed so efficiently as to maintain low administered concentrations. More efficient delivery should reduce the number of cells exposed, favour uptake, and reduce the potential for off-target effects, hence maintaining a suitable therapeutic index.

## 6. CONCLUSIONS

Although the field of rnai is still an emerging one, the benefits of understanding the roles of mirnas and sirnas in cancer are potentially enormous, not only for their roles as cancer loci, but also because for the growing likelihood that small rnas can be used as therapeutics in treating cancer itself 30.
